# Gastrin and Nitric Oxide Production in Cultured Gastric Antral Mucosa Are Altered in Response to a Gastric Digest of a Dietary Supplement

**DOI:** 10.3389/fvets.2021.684203

**Published:** 2021-10-04

**Authors:** Jennifer L. MacNicol, Wendy Pearson

**Affiliations:** Department of Animal Biosciences, Ontario Agricultural College, University of Guelph, Guelph, ON, Canada

**Keywords:** *in vitro*, tissue culture, gastrin, nitric oxide, interleukin-1β, dietary supplement, gastric, stomach

## Abstract

*In vitro* organ culture can provide insight into isolated mucosal responses to particular environmental stimuli. The objective of the present study was to investigate the impact of a prolonged culturing time as well as the addition of acidic gastric fluid into the *in vitro* environment of cultured gastric antral tissue to evaluate how altering the commonly used neutral environment impacted tissue. Furthermore, we aimed to investigate the impact of G's Formula, a dietary supplement for horses, on the secretion of gastrin, interleukin1-beta (IL-1β), and nitric oxide (NO). These biomarkers are of interest due to their effects on gastric motility and mucosal activity. Gastric mucosal tissue explants from porcine stomachs were cultured in the presence of a simulated gastric fluid (BL, *n* = 14), simulated gastric fluid containing the dietary supplement G's Formula (DF, *n* = 12), or an equal volume of phosphate buffered saline (CO, *n* = 14). At 48 and 60 h, 10^−5^ M carbachol was used to stimulate gastrin secretion. Cell viability was assessed at 72 h using calcein and ethidium-homodimer 1 staining. Media was analyzed for gastrin, IL-1β, and NO at 48, 60, and 72 h. There were no effects of treatment or carbachol stimulation on explant cell viability. Carbachol resulted in a significant increase in gastrin concentration in CO and DF treatments, but not in BL. NO was higher in CO than in BL, and NO increased in the CO and DF treatments but not in BL. In conclusion, the addition of carbachol and gastric digests to culture media did not impact cell viability. The use of an acidic gastric digest (BL) reduced the effect of cholinergic stimulation with carbachol at a concentration of 10^−5^ M and reduced NO secretion. The addition of the dietary supplement to the gastric digest (DF) appeared to mediate these effects within this model. Further research is required to evaluate the specific effects of this dietary supplement on direct markers of mucosal activity and the functional relevance of these results *in vivo*.

## Introduction

Gastric motility is a strictly regulated physiological function that relies on physical, chemical, and neurological signals to integrate information from the gastric environment. Dysregulation of any of the integrated signals can result in gastric motility disorders. Furthermore, there are neurological and hormonal mechanisms that integrate signals from distal regions of the gastrointestinal (GI) tract that significantly influence gastric motility *in vivo*. Ileal break is a principal nutrient-triggered control mechanism that acts to slow gastric motility following the ingestion of a meal (1). This feedback mechanism relies on signals from the distal small intestine that act to delay gastric emptying, thus enabling improved nutrient absorption. Infusion of short-chain fatty acids into the distal ileum can induce gastroparesia (2, 3), which has been linked to humoral control involving peptide YY (4). In horses, inappropriate GI motility has been associated with several critical conditions including colic, obstructive disorders, and ileus (5). Nutritional options for optimizing equine gastric health using dietary feed supplements and additives are appealing alternatives to pharmaceutical treatment of GI disorders.

G's Formula^™^ (GF), manufactured by G's Organic Solutions INC. (BC, CANADA), is an equine dietary supplement formulated to optimize equine GI health. It is composed of whole food ingredients including dried cabbage, carrot, oat flour, and hemp. Both cabbage (*Brassica oleracea* var. *capitata*) and carrot (*Daucus carota L*.) have demonstrated anti-ulcerogenic, analgesic, and anti-inflammatory properties, and have historically been used to enhance and maintain GI health (6, 7). Beta-glucans, polysaccharides in which β-glucosidic bonds link glucose monomers, are prevalent in cereals such as wheat, oats, and barley. The antioxidant activity and immunomodulatory characteristics of beta-glucans contribute to their positive health benefits (8). The combination of ingredients in GF were selected based on their historical use in promoting and maintaining GI health.

The stomach has a pH of ~2 (9). Therefore, culturing mucosa in an acidic environment may create a model that is more reflective of the physiological conditions to which the gastric mucosa is commonly exposed. The hormone gastrin, secreted by G cells in the pyloric antrum of the stomach, is an important stimulus for gastric acid secretion and promotes epithelial cell proliferation (10). While increased gastric pH promotes gastrin secretion, the presence of gastric acid in the stomach stimulates antral D cells to secrete somatostatin (St), which, in a negative feedback mechanism, inhibits G-cell secretion of gastrin (10). Other biomarkers of interest involved in regulating the gastric environment include nitric oxide (NO) and interleukin 1-beta (IL-1β). Both these signals have been reported to have protective and deleterious impact on the ability of the gastric mucosa to withstand damage from luminal stimuli and acid (11). Exposure of tissue to a stimulated environment provides a means to evaluate feedback systems that exist between these biomarkers. Furthermore, gastric smooth muscle cells contain gastrin receptors (12), and both exogenous (13) and endogenous (14) gastrin have been found to influence gastric motility. NO is an important molecular signal within the GI tract. Low concentrations of NO are crucial for maintaining mucosal defenses such as epithelial barrier function and microcirculation (15). NO is involved in the control of GI motility via inhibitory pathways, acting directly as a stimulus for inhibitory neurotransmitter release and indirectly as inhibitor of stimulatory neurotransmitters (16). IL-1β released by the rat stomach retards gastric emptying (17, 18). It has been reported to potentiate CaCl_2_-induced contractions in rat isolated stomach smooth muscle strips (19), as well as inhibit acetylcholine (Ach)-induced mechanical activity of GI smooth muscle (20). The influence of a simulated digest of GF on the response of gastric smooth muscle to Ach has been previously investigated by our lab (21). A simulated digest of GF increases the contractility of gastric smooth muscle in response to increasing concentrations of Ach *in vitro*. However, the initial exposure to any feed within the GI tract is the gastric mucosa.

The objective of the current study was to evaluate the influence of acidic gastric digests on the secretory activity of the pyloric antrum in an *in vitro* organ culture model. Furthermore, the effects of a gastric digest of GF on gastric mucosal secretions of biomarkers relevant to GI smooth muscle contractility and GI motility *in vitro* were investigated. It was predicted that tissue cultured with acidic digests would display a different secretory profile than tissue cultured without the use of digests. Furthermore, the use of GF would further alter levels of gastrin, IL-1β, and NO within culture media.

## Materials and Methods

All reagents were purchased from Sigma Aldrich Canada unless otherwise stated. All spectrophotometric readings were obtained using a Victor^3^ plate reader (PerkinElmer, USA).

### Experiment 1: Tissue Viability in Culture

The objective of this initial experiment was to determine the effect of 120 h of culture on viability of porcine antral mucosa explants in order to determine an optimal culture timeframe. A total of 36 porcine antral explants were obtained from the antral stomach of 3 pigs (12 explants per pig). Initial viability was assessed on 18 fresh explants immediately following specimen dissection. Subsequently, four to six explants were removed from culture at 6, 24, 48, 72, 96, and 120 h, and viability was assessed using differential calcein AM and ethidium homodimer-1 (Eth-D) staining (see below for details).

#### Tissue Collection and Culture

Porcine stomachs (*n* = 3) were collected from a local abattoir (Reist and Weber, St. Jacobs, ON) and transported on ice in phosphate buffered saline (PBS). Stomachs were opened along the lesser curvature and a section of pyloric antral tissue was excised (~5 × 5 cm). The entire mucosal surface, comprising the luminal epithelial cells and basolateral cells (9), was separated from the underlying smooth muscle using blunt dissection. Mucosal tissue was gently rinsed with PBS to remove adherent particulates and then pinned taunt onto a silicone bottom dish. All of the following procedures were performed using aseptic conditions in a laminar flow hood. Tissue explants were obtained using a sterile, 4-mm punch biopsy (~0.014 g). Each explant was rinsed twice in sterile culture media [Dulbecco's Modified Eagle Medium (DMEM) containing 100 U/L penicillin and 100 μl/L streptomycin, 1 ml/L amphotericin, 5% fetal bovine serum (GE Healthcare, HyClone, CAN)]. Single explants were placed in each well of a sterile 24-well culture plate (Primaria, Corning, NY). Two milliliters of culture media with 10 μl/ml freshly prepared glutamine was added to each well. Culture dishes were covered and placed in an incubator at 37°C (7% CO_2_) for up to 120 h. Media was aspirated and refreshed initially at 6 h, and then every 24 h.

#### Viability Assessment

The viability of mucosal cells was assessed using the LIVE/DEAD^®^ Viability/Cytotoxicity Kit (Invitrogen^™^, CA) according to the manufacturer's instructions for fluorescence measurements using a microplate reader. In order to optimize dye concentrations and incubation time, as per the manufacturer's instructions, tissue explants obtained by 4-mm punch biopsy with a thickness of ~3 mm were utilized. A positive control composed of 18 fresh explants (six explants per pig from three pigs) and a corresponding negative control of 18 dead explants (six explants per pig), in which cells were killed by a 30-min incubation in anhydrous ethanol, were utilized. In brief, cells were stained with calcein AM (10 μM) and Eth-D (20 μM) and incubated at room temperature (60 min). The microplate reader was set to scan each well, beginning at the bottom, with 10 horizontal steps at each of three vertical displacements set 0.1 mm apart. This was done to generate measurements of fluorescence at 30 points at three depths across the explant. The calcein AM fluorescence of live cells and EthD-1 fluorescence of dead or damaged cells in explants was measured with excitation and emission filters of 485 and 530 nm and 530 and 685 nm, respectively. The Invitrogen^™^ LIVE/DEAD^®^ Viability/Cytotoxicity Kit for Mammalian cells takes advantage of differential stain penetration of cells based on membrane integrity and their different fluorescence profiles. Calcein penetrates live cells and indicates intracellular esterase activity, while Eth-D is permeable only to cells with damaged membranes. Percent live cells were calculated as: (live cells)/(live cells + dead cells) ^*^ 100.

#### Statistics

Data were analyzed using PROC GLIMMIX in SAS 9.4 (SAS Institute Inc., NC, USA). The following model was used to assess percent viability (y):


$$\begin{array}{l} y_{\text{ij}}=\mu +pig_{\text{i}}+time_{\text{j}}+\varepsilon_{\text{ij}} \end{array}$$

where *μ* = the overall mean, pig = the random effect of the pig (i = 1–3), time = fixed effect of time point (j = 0–120 h), and ε = the experimental error. Least square means using a Tukey adjustment were calculated. Data are presented as mean ± SEM and *p* ≤ 0.05 was considered significant.

### Experiment 2: Effects of Gastric Digests on Antral Mucosal Tissue Secretions

This experiment was performed to determine the influence of a gastric digest of the dietary supplement G's Formula^™^ (DF) on porcine antral explants *in vitro*. A blank gastric digest (BL), without the addition of the GF, was included to evaluate the effect of the digest procedure components on explant viability and secretion. A PBS control (CO) was included to evaluate the influence of time on explants. Carbachol (10^−5^ M) was used to stimulate gastrin secretion from tissue explants. The dose of carbachol used was based on maximal stimulation of gastrin as demonstrated in previous studies (22–26).

Prolonged culture of gastric mucosa is notoriously difficult due to challenges with microbial contamination. In the first set of experiments, tissue viability remained statistically consistent at ~94% between 48 and 72 h. Therefore, we chose to reduce the timeline to 72 h, exposing the tissue to carbachol stimulation at 48 and 60 h. Culture media was assayed for gastrin, NO, and IL-1β as detailed below.

A total of 14 stomachs from market weight pigs were used. Experiments were run using four pigs per experimental run (a total of four experimental runs were completed). The final run included only two pigs. Explants from the stomach of each pig were run through all treatments and stimulations and therefore animal was used as a random effect in the statistical analysis to account for secretory differences between individuals.

#### Experimental Design

In order to determine an impact of treatment, stimulation by carbachol, and time, between and within treatments, explants from the same animal were exposed to the following conditions: G's formula (DF) stimulated with carbachol or PBS (DF_stim, DF_non); a blank digest, to assess the impact of the digest without the inclusion of G's formula on mucosal secretions, stimulated with carbachol or PBS (BL_stim, BL_non); a PBS control, to assess the impact of time and the carbachol model on secretory activity, stimulated with carbachol or PBS (CO_stim, CO_non).

A 24-h culture period was undertaken to allow samples to equilibrate to culture conditions after excision (27–30). Gastrin data from a subsample of wells were analyzed during the viability pilot and demonstrated a rise in media gastrin by 24 h, which stabilized by 48 h and remained consistent until the end of a 5-day culture period. Harty et al. (31) also demonstrated a progressive rise in media gastrin over a 24-h culture period. A 24-h period in which samples were exposed to simulated digest treatments was employed as a means of conditioning explants to the treatment conditions. This was decided in order to avoid potentially confounding treatment effects with a tissue response to an initial disturbance of culture conditions. A media refreshment during this 24-h period was decided against in order to reduce the chance of contamination. The viability of samples demonstrated a stabilization between 48 and 72 h and therefore within this period was determined to be the optimal time to stimulate. Consecutive stimulations with carbachol were undertaken as a means of enhancing gastrin release so as to amplify potential differences in response. As pilot data (not shown) had demonstrated a significant rise in media gastrin 12 h following stimulation, 60 h was used as the second stimulation time point.

**Figure 1:**
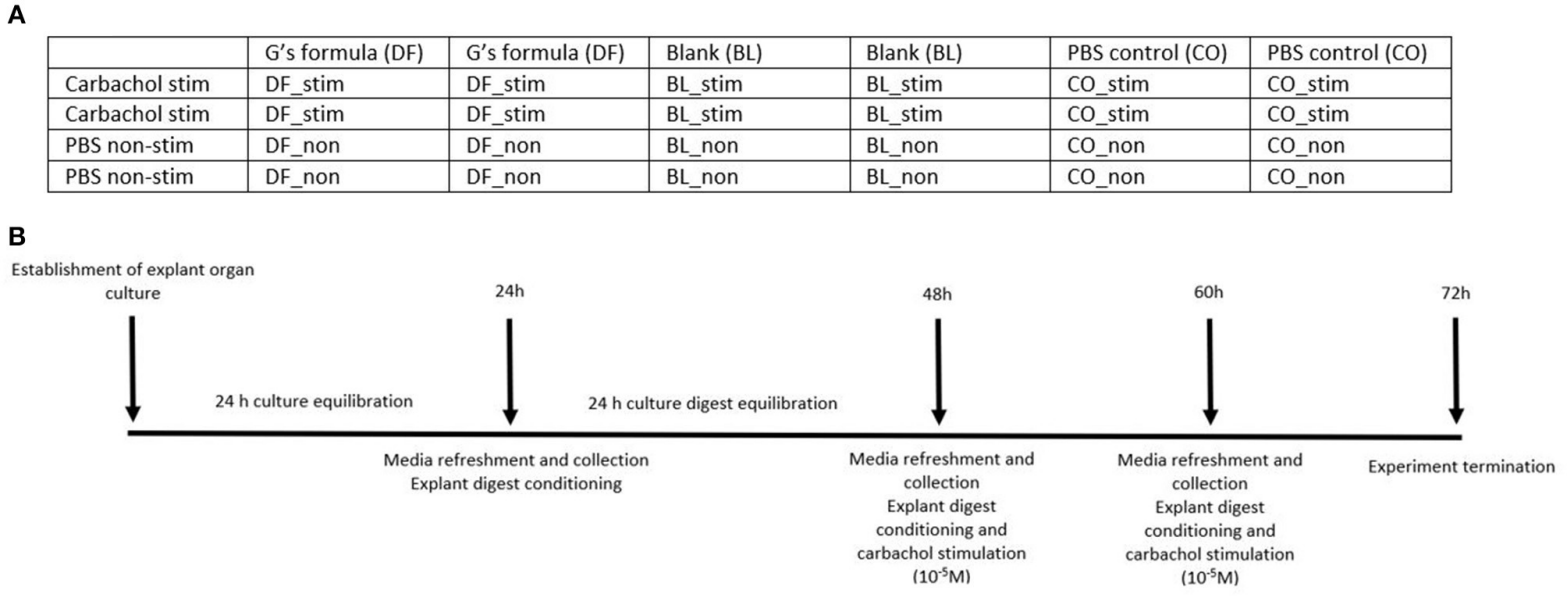
**(A)** A plate map of the treatments used on a culture plate. BL_non, explants were treated with a blank gastric digest and stimulated with sterile PBS; BL_stim, explants were treated with a blank gastric digest and stimulated with 10^−5^ M carbachol; CO_non, explants were treated with sterile PBS and stimulated with sterile PBS; CO_stim, explants were treated with sterile PBS and stimulated with 10^−5^ M carbachol; DF_non, explants were treated with a gastric digest of the dietary supplement G's Formula^™^, composed of dried cabbage, carrot, hemp, and oat flour, and stimulated with sterile PBS; DF_stim, explants were treated with a gastric digest of the dietary supplement G's Formula^™^ composed of dried cabbage, carrot, hemp, and oat flour, and stimulated with 10^−5^ M carbachol. **(B)** A visual timeline of the experimental protocol.

Figure 1A represents a plate map of a culture plate used in this study. The series of stimulation and treatments was randomized between plates. Figure 1B represents a visual timeline of the experimental protocol.

#### Simulated Digestion Protocol

To better reflect the acidic conditions within which the gastric mucosa functions and to which it would be exposed, a simulated gastric digest of GF was made. The protocol used was the gastric phase of digestion adapted from a simulated digestion protocol used to generate consistent digests of feedstuffs for *in vitro* tissue exposure (21, 27–29). In brief, 160 mg/ml of supplement was added to simulated gastric fluid composed of 2.16 g/L 3 mM NaCl, 30 ml/L 0.03N HCl, and 3.2 mg/ml porcine pepsin (added fresh per use). The gastric digest was incubated at 37°C in a water bath shaker for 2 h. Following incubation, the gastric digest was serially filtered using a perforated funnel filter, 0.45-μm syringe filter unit (Millex-HA, Millipore, ON), and 0.22-μm syringe filter unit (Millex-GP, Millipore, ON) into sterile tubes (Thermo Scientific, Thermo Fisher Scientific, CAN) and kept at 4°C. The g of dietary supplement used was calculated such that the concentration in the 1.5-ml volume of media was reflective of a dosage of a total daily dose of 160 g of supplement. The manufacturer's recommended dose for a 450-kg horse is 80 g 2×/day. Therefore, calculations were based on 160 g in 10-L volume [the approximate volume of the equine stomach (32)]. The BL gastric digest was made using the same protocol without the added dietary supplement. The pH of the working DF gastric digest was ~2.62 and that of the BL gastric digest was ~2.32.

#### Tissue Collection and Culture

Tissue explants were obtained from porcine stomachs (*n* = 14), and preparation methods were as stated previously with the following modifications to reduce the potential for contamination. Mucosal tissue was rinsed with de-ionized water to remove any adherent particulates prior to being pinned onto a silicone bottom dish and bathed in PBS, 100 U/L penicillin and 100 μl/L streptomycin, and 1 ml/L amphotericin. In a sterile culture hood under aseptic conditions, explants were rinsed twice in the PBS with antibiotic. Two explants were placed in each well of sterile 24-well culture dishes with 1.5 ml of culture media added per well. A total of 24 explants per stomach were cultured. Following a 24-h equilibration period, media was aspirated and replaced with 1.4 ml of media with an additional 100 μl of either DF, BL, or CO. This process was repeated at 48 h with the addition of each treatment being stimulated by either carbachol (10^−5^ M; EMD Millipore Corp., USA; DF_stim, BL_stim, CO_stim) or sterile PBS (DF_non, BL_non, CO_non). This concentration of carbachol was chosen based on previous dose–response studies of carbachol on culture media gastrin to generate a repeatable and maximal response (22, 23). Media was refreshed and digest/stimulation was repeated at 60 h. The experiment was terminated at 72 h. Subsequent experiment termination explants were weighed, and viability was assessed (see below). Aspirated media was stored at −20°C until analyzed.

#### Viability Assessment

Cell viability of explants from the first eight porcine stomachs was measured following the termination of the experiment, as previously described. These data were assessed in PROC GLIMMIX SAS 9.4 (SAS Institute Inc., NC, USA) using a model with the random effect of pig, fixed effect of treatment (CO, BL, or DF), fixed effect of stimulation (stim or non-stim), and interaction between treatment and stimulation. The difference between least square means using a Tukey adjustment was assessed and *p* ≤ 0.05 was considered significant. Based on this analysis, it was concluded that neither treatment nor stimulation significantly affected cell viability and therefore further viability assessments were discontinued.

#### Measurement of Media Gastrin and IL-1β Concentrations

Media gastrin and IL-1β were measured using porcine specific gastrin (MyBiosource Inc, SD, CS; CAT# MBS736639; intra-assay SD ±0.1, interassay SD ±0.6) and IL-1β (MyBioSource Inc, SD, CA; CAT# MBS260684; intra-assay SD ±0.04, interassay SD ±0.05) ELISA kits according to the manufacturer's instructions.

#### Measurement of Media Nitrite

Media nitrite, a stable oxidation product of NO, was measured using the Griess reaction adapted for measurement by spectrophotometric analysis (33). In brief, samples were added to 96-well plates in duplicate. Sulfanilamide (0.01 g/ml), N-(1-Napthyl)ethylenediamide dihydrochloride (0.001 g/ml), and phosphoric acid (0.25 ml/ml) were added to all wells. Absorbance was read immediately at 530 nm. Sodium nitrate was used to generate a standard curve on each plate, to which sample absorbances were compared to calculate sample concentrations (intra-assay SD ±0.003, interassay SD ±0.02).

#### Statistics

Media concentrations of biomarkers were analyzed using PROC GLIMMIX in SAS 9.4 (SAS Institute Inc., NC, USA). Concentrations were standardized per gram of mucosal tissue wet weight (y). A RM ANOVA was used according to the following model:


$$\begin{array}{l} y_{\text{hijkl}}=\mu +\beta xinitial+pig_{\text{i}}+trt_{\text{j}}+stim_{\text{k}}+time_{\text{l}}+trt_{\text{j}}^{*}stim_{\text{k}}\\ \;+trt_{\text{j}}^{*}time_{\text{l}}+stim_{\text{k}}^{*}time_{\text{l}}+trt_{\text{j}}^{*}stim_{\text{k}}^{*}time_{\text{l}}+\varepsilon_{\text{ijkl}} \end{array}$$

where *μ* = the overall mean, *β* = the covariate slope, initial = the concentration/g tissue at 48 h, pig = the random effect of the pig (i = 1–14), txt = the fixed effect of treatment (j = CO, BL, and DF), stim = the fixed effect of stimulation (k = stim, non-stim), time = the repeated measure of the h sampled (l = 48, 60, and 72), all interactions of fixed effects were included, and *ε* = the experimental error. The residuals of different covariance structures were analyzed to identify the most appropriate structure, and lognormal distributions were used to improve normality if required. Least square means were used to analyze the difference between treatment, stimulation, and time; least square mean interactions sliced by effect were used to analyze the differences between the various levels of effects. Data are presented as means ± SEM/g tissue wet weight unless otherwise specified and *p* ≤ 0.05 was considered significant.

This design and statistical methodology enable the overall differentiation between the type III main effects of time, treatment, and stimulation. It also permits differentiation within treatments over time.

## Results

### Experiment 1: Cell Viability

Explant viability remained high throughout the 120-h culture period (97.5% ± 0.16–92.6% ± 0.38). A significant effect of time on cell viability (*p* < 0.001) was observed (Table 1).

**Table 1:** Porcine stomachs (*n* = 3) were collected from a local abattoir and 4-mm punch biopsies of antral mucosal explants were cultured over 120 h in sterile DMEM containing 100 U/L penicillin and 100 μl/L streptomycin, 1 ml/L amphotericin, and 5% fetal bovine serum that was refreshed daily.

**Hour**	**% Viability**	**SEM**
0	97.5^a^	0.16
6	97.4^a^	0.11
24	95.6^b^	0.23
48	94.4^c^	0.23
72	94.5^c^	0.22
96	93.4^bcd^	0.69
120	92.6^d^	0.38

*% Viability was assessed by calcein (10 μM) and ethidium homodimer-1 (20 μM) staining at 0, 6, 24, 48, 72, 96, and 120 h*.

*Different superscripts represent time points that are significantly different (p ≤0.05)*.

### Experiment 2: Effects of GF on Mucosal Tissue Secretions

#### Cell Viability

**Figure 2:**
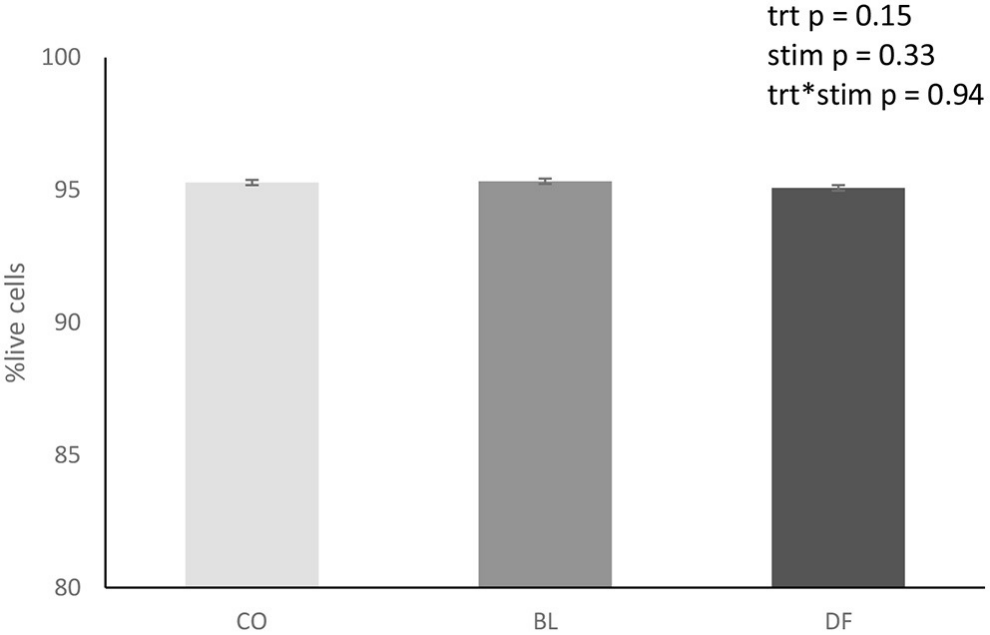
Overall treatment viability of antral mucosal explants ± SEM collected from porcine stomachs (*n* = 8) and cultured for 72 h in sterile DMEM containing 100 U/L penicillin and 100 μl/L streptomycin, 1 ml/L amphotericin, and 5% fetal bovine serum that was refreshed daily. Viability was assessed by calcein (10 μM) and ethidium homodimer-1 (20 μM) staining at 72 h. CO (CO_stim and CO_non; *n* = 14) explants treated with sterile PBS. BL (BL_stim and BL_non; *n* = 14), explants treated with a blank gastric digest. DF (DF_stim and DF_non; *n* = 12), explants treated with a simulated gastric digest of G's Formula^™^ composed of dried cabbage, carrot, hemp, and oat flour. There was no significant effect of treatment, stimulation, or treatment by stimulation interactions.

Viability at 72 h was between 95.1 and 95.3% ± 0.01 in all treatments. There was no effect of treatment (*p* = 0.15; Figure 2) or carbachol stimulation (*p* = 0.33) on explant cell viability.

#### Media Gastrin Concentration

There were no main effects of time (*p* = 0.12) or treatment (*p* = 0.06) on media gastrin. In explants stimulated with carbachol, gastrin increased over time in CO from 48 h (*p* = 0.03) and 60 h (*p* = 0.05) to 72 h. Similarly, in DF-conditioned explants, gastrin increased over time from 48 to 60 h (*p* = 0.05). No differences were observed over time in BL-conditioned explants (Table 2). Supplementary Data includes gastrin concentrations not standardized per g tissue ww.

**Table 2:** Media gastrin (pg/ml/g tissue ww) as assessed by spectrophotometric assay of a commercial ELISA for porcine gastrin over time in porcine antral explants stimulated with either 10^−5^ M carbachol (_stim) or sterile PBS (_non).

**Treatment**	**Hour**	**Gastrin (pg/ml/g tissue ww)**	**SEM**
CO_stim	48	12,379^a^	1,256.5
	60	13,098^a^	1,329.4
	72	16,426^b^	1,492.8
			
CO_non	48	13,171	1,397.3
	60	11,557	1,226.1
	72	13,433	1,492.8
			
BL_stim	48	13,718	1,333.3
	60	11,626	1,130.0
	72	13,890	1,350.0
			
BL_non	48	13,563	1,264.8
	60	12,569	1,172.1
	72	13,335	1,243.6
			
DF_stim	48	12,787^a^	1,241.0
	60	15,915^b^	1,544.5
	72	16,303^ab^	1,582.2
			
DF_non	48	14,383	1,609.6
	60	14,738	1,649.4
	72	14,248	1,594.5

*Explants were taken with 4-mm punch biopsy and cultured in sterile DMEM containing 100 U/L penicillin and 100 μl/L streptomycin, 1 ml/L amphotericin, and 5% fetal bovine serum that was refreshed daily. CO_stim (n = 10)/CO_non (n = 10), explants treated with sterile PBS. BL_stim (n = 10)/BL_non (n = 13), explants treated with a blank gastric digest. DF_stim (n = 11)/DF_non (n = 9), explants treated with a simulated gastric digest of G's Formula^™^, composed of dried cabbage, carrot, hemp, and oat flour*.

*Different superscripts represent time points that are significantly different within treatments (p ≤0.05)*.

#### Media IL-1β Concentration

There were no main effects of treatment (*p* = 0.73) or stimulation (*p* = 0.75) on media *IL-1*β. There was a significant overall effect of time on media IL-1β (*p* = 0.0005). The concentration of media IL-1β increased over time from 48 h (1,905 ± 42.4 ng/ml/g ww) to 60 h (2,078 ± 42.34 ng/ml/g ww; *p* = 0.0002) and 72 h (2,038 ± 42.4 ng/ml/g ww; *p* = 0.004).

#### Media Nitrite Concentration

There was no significant effect of stimulation (*p* = 0.11) on media nitrite. There were overall effects of treatment on media nitrite, which was lower in the CO (458 ± 6.8 μM/ml/g ww) compared to BL-conditioned (475 ± 7.4 μM/ml/g ww; *p* = 0.03).

Overall media nitrite decreased over time in the CO explants from 48 to 60 h (*p* = 0.0008) and 72 h (*p* < 0.0001). Similarly, in DF-conditioned explants, nitrite decreased over time from 48 to 72 h (*p* = 0.007). There was no effect of time on media nitrite in BL-conditioned explants (Table 3).

**Table 3:** Overall media nitrite (μM/ml/g tissue ww) as assessed by spectrophotometric assay of the Griess reaction over time in porcine 4-mm punch biopsy antral explants cultured in sterile DMEM containing 100 U/L penicillin and 100 μl/L streptomycin, 1 ml/L amphotericin, and 5% fetal bovine serum and refreshed daily.

**Overall treatment**	**Hour**	**Media nitrite (μM/ml/g tissue ww)**	**SEM**
CO	48	481^a^	8.6
	60	452^b^	8.2
	72	442^b^	8.1
			
BL	48	484	9.5
	60	470	9.2
	72	471	9.2
			
DF	48	488^a^	10.6
	60	472^ab^	10
	72	459^b^	9.8

*CO (CO_stim and CO_non; n = 14), explants treated with sterile PBS. BL (BL_stim and BL_non; n = 14), explants treated with a blank gastric digest. DF (DF_stim and DF_non n = 12), explants treated with a simulated gastric digest of Gs Formula^™^, composed of dried cabbage, carrot, hemp, and oat flour*.

*Different superscripts represent time points that are significantly different within treatments (p ≤0.05)*.

## Discussion

The antral organ culture protocol used in this study took advantage of a simulated digestion procedure (21, 27–29) to generate a gastric digest of GF. Overall cell viability was not impacted by the addition of DF to culture media during the 72-h culture period. Furthermore, there was an increase in gastrin secretion over time in CO and DF explants stimulated with carbachol, and in NO secretion over time in combined stimulated/unstimulated CO and DF explants, responses that were not observed in BL.

Full thickness organ culture techniques represent an appealing option for targeted evaluations into integrated gastric mucosal secretory activity. *In vivo* research of isolated mucosal activity is challenging due to the confounding variables present within a complete system. Both horses and pigs are monogastric species that have composite stomachs (34). Pyloric glands residing in the antrum of the stomach contain gastrin-secreting G cells, St-secreting D cells, and mucus-secreting cells (9, 35). Histological staining techniques have identified mucous, chief, St, and gastrin cells within the distal portion of the porcine stomach (36). Similarly, the distal portion of the equine stomach is the site of hormone and mucus secretion and immunohistochemical analysis has also identified these cell types within this region (37). Due to the structural and cellular similarities between the porcine and equine stomach (38–40), the use of porcine tissue represents a practical alternative to the collection of equine gastric tissue, which can be difficult due to accessibility, the lack of homogeneity across subjects, and challenges with the influence of sedatives and euthanasia drugs on tissue physiology.

Historically, gastric antral organ culture evaluating the actions of hormones on gastric tissue have been performed for limited periods of time at neutral pH (22, 25, 26, 31, 41). However, the stomach has a pH of ~2 (9). Therefore, in this study, tissue was exposed to a gastric digest of our dietary supplement of interest so as to create a model that better represented the physiological conditions to which the luminal surface of the gastric mucosa is exposed. A high level of cell viability was maintained as measured through calcein/Eth-D staining despite exposure to acidic or carbachol-stimulated culture conditions. Our results are consistent with others, who report that porcine gastric tissue remains viable in culture for up to 10 days, as determined by histochemical analysis (42). Calcein AM and Eth-D differential staining has been used as a means of viability assessment in full thickness cartilage explants (28–30) and as a viability parameter in the assessment of uterine tissue pieces (43, 44). Therefore, the quantitative and repeatable means of assessing cell viability provided by a differential staining assay is considered an appropriate preliminary method to demonstrate the viability of cells within this system. However, histological evaluations or the use of confocal laser scanning microscopy would have provided a more detailed evaluation of tissue cellular shape and distribution within the tissue. Further analysis of mucosal defensive mechanisms active within this model is warranted to identify factors that contribute to the maintenance of such high levels of cell viability. Morphological assessments could be included in future investigations and would be particularly informative if mucosal culturing were to be used in a disease model.

An important limitation of this study and consideration when interpreting results is that, despite the use of an acidic digest, the culture conditions had noteworthy distinctions from *in vivo* gastric physiology. The exposure of tissue to gastric digests chosen for this study is substantially longer than typical gastric retention. Seventy-five percent of a fluid marker exits the equine stomach within 30 min (45). The full digestive period is generally considered to be between 2 and 3 h following a meal (46). Therefore, exposing tissue to a digest for 12 h represents a prolonged exposure. Whether the use of a shorter period of exposure would have demonstrated a heightened or reduced response to treatment is worthy of further exploration and would provide relevant details regarding the influence of this particular dietary supplement. Currently, the results should be interpreted within the context of the particular set of culture conditions utilized in this experiment.

In the current study, the cholinergic agonist carbachol was used to stimulate antral G cells to secrete gastrin. Cholinergic stimulation of antral tissue by carbachol results in a sustained and progressive increase in gastrin release over time (47). In CO-stim explants, cholinergic stimulation significantly enhanced gastrin secretion, which is consistent with other studies (22–25, 47). Creating an acidic culture environment through the use of a blank acidic digest in our model (BL-stim) abolished the stimulatory effect of carbachol on gastrin release. Cholinergic stimulation of gastrin is mediated in part by St inhibition (25). St is a potent inhibitor of gastric acid secretion from the stomach that acts both directly on acid-secreting parietal cells and indirectly through the inhibition of gastrin secretion from G cells and histamine secretion from enterochromaffin-like cells (ECL) (48). It is possible that a reduction in pH invoked by the acidic conditions created by the blank digest stimulated St release, countering the influence of cholinergic stimulation on gastrin release.

Interestingly, in contrast to the effect of BL, when exposed to DF, cholinergic stimulation remained effective in stimulating gastrin release. When gastric smooth muscle is exposed to a DF, it increases Ach induced contractility (21). It was proposed that the potentially synergistic actions of the flavonoid components of GF, contributed to by the vegetable whole ingredients used in the product, sensitize smooth muscle cells to cholinergic stimulation. It is possible that a sensitizing effect to cholinergic stimulation is also responsible for the maintenance of gastrin synthesis by the mucosa in this model. As the main function of gastrin in the stomach is a stimulus of acid secretion, the use of ^14^C aminopyrine uptake from media would provide insight into the functional impact of DF-induced gastrin secretion. No dose–response curve to carbachol in the particular culture conditions used in this study was undertaken, and only 10^−5^ M carbachol was used to stimulate tissue. Therefore, it is difficult to ascertain how the digest might have impacted tissue sensitivity to various doses of carbachol *in vitro*. Hence, any conclusions regarding the sensitizing effects of G's formula cannot be extrapolated beyond the dose of carbachol used in the current study. Furthermore, media gastrin represents the sum of gastrin from several sources and is not specific to actively stimulated gastrin release. As we only measured media gastrin, it was not possible to differentiate between gastrin sources. Assessment of mucosal gastrin would have aided in deciphering whether there was an impact on gastrin synthesis, secretion, or both. Additionally, an alteration in St synthesis or release resulting from tissue exposure to DF may also have been involved in the maintenance of gastrin secretion. St was not measured in this study, in part because carbachol has been found not to significantly alter St release in antral mucosa organ culture (24). However, antral mucosa cultured with an acidic gastric digest may demonstrate different dynamics than what is observed at neutral pH and future studies should consider analyzing media for both gastrin and St.

A time-dependent increase in media IL-1β was noted in this study that was not influenced by either cholinergic stimulation or treatment with a gastric digest. Similarly, a study by Montuschi et al. (49) demonstrated a time-dependent release of IL-1β from the rat gastric fundus that was not altered by treatments that induce IL-1β synthesis or electrical field stimulation. The function of the time-dependent release of IL-1β *in vitro* is unclear. Exogenous IL-1β dose dependently inhibits carbachol-stimulated acid secretion from parietal cells (50). It is also protective against GI damage caused by ethanol, indomethacin, cysteamine, and aspirin (51, 52). In the current study, the consistent increase in media IL-1β observed, regardless of treatment or stimulation, suggests its involvement in a protective mechanism. Although it is appealing to speculate that IL-1β may be contributing to mucosal defense against the mild irritation caused by media aspiration and refreshment, no quantitative measures of mucosal defense, such as mucus secretion, epithelial barrier function, or leukocyte adherence, were examined in the current study. Therefore, it is possible that IL-1β was acting in a pathological manner.

Interleukin-1 beta and NO exhibit integrated regulation in several biological systems. We observed an inverse relationship between IL-1β and NO over time, with an increase in IL-1β mirroring a decrease in NO, which was apparent in CO and DF. Interleukin-1 beta stimulates inducible nitric oxide synthase (iNOS) in several different cell types including cardiocytes (53), pancreatic-beta cells (54), and chondrocytes (55). Conversely, iNOS inhibition results in an increase in IL-1β protein secretion and NO-producing compounds inhibit IL-1β release from macrophages (56, 57). It is possible that, in this study, the reduction in NO over time facilitated the increase in IL-1β from the gastric mucosa. However, the increase in IL-1β was apparent in all treatments, whereas the reduction in NO was not, which suggests that these systems did not directly co-regulate each other in this model.

Positive controls for IL-1β or NO were not included in this study. The stimulation of gastrin through the use of carbachol has the strongest evidence for repeatable and consistent results when used in porcine antral organ culture within the literature. Therefore, we choose to develop our model using carbachol as the primary stimulus. However, future studies investigating the mechanisms of action and impacts on mucosal function of inflammatory or free radical regulators should include positive controls of these biomarkers and evaluate culture media levels of these positive controls to ensure that the model reacts as expected. Nevertheless, based on the reaction of the model to carbachol, similar results seen within other gastric mucosal studies on IL-1β concentrations over time (49), and the substantial role of NO within the stomach on mucosal acid (58, 59) and mucus production (60), we believe that the results noted in this study on IL-1β and NO represent true mucosal secretory responses.

An additional consideration to the interpretation of the decrease in media concentration of NO overtime in the CO treatment is that neutral conditions are not truly reflective of the gastric environment. Gastric mucosa is most commonly cultured at a neutral pH (24, 41, 42, 61). However, in the majority of mammals, gastric pH can be as low as 2 (62). This is of significance when interpreting results that demonstrate changes in gastric function. Mucosal activity in neutral culture might be considerably different than gastric responses at physiological pH. Our result regarding the difference in NO release between CO and BL explants highlights this discrepancy. The CO explants had pH neutral PBS added to the media, whereas BL explants had a digest with a pH of 2 added to the media. NO plays a role in gastric acid regulation and mucus secretion (58, 63). It is possible that the impact of NO on gastric acid and mucosal defense mechanisms was reflected in the differential concentrations of media NO between CO and BL. However, this response was not investigated in detail and was beyond the scope of this study. Nevertheless, this differential response highlights the importance of conservative data interpretation in culture studies, particularly because mucosal response can be highly influenced by culture conditions. Culture conditions that are more reflective of the luminal environment to which tissue is exposed generate results that are more likely to relate to *in vivo* tissue responses.

A limitation to the interpretation of the G's Formula results is that, although a blank-simulated digest was included, none of the specific component parts, bioactive compounds, or osmolar effects of G's Formula on the digest were evaluated in the model. Rather, we choose to evaluate a digest of the product in its entirety so as to capture the effects of the complete product. Although it would be of interest to determine which specific ingredients or compounds within the product have the greatest impact on gastric hormone secretion, we were more interested in the influence of the combination of ingredients. Therefore, we choose to utilize a whole product-simulated digest. Additionally, it can be challenging to discern individual components of whole food products that impact physiological function as many bioactive compounds display synergistic or antagonistic activity (64, 65) and therefore do not demonstrate particular effects when isolated. Nevertheless, more digests of particular factions of G's Formula would provide superior insight into how this product may influence the mucosa.

Although this study was investigating a supplement designed for horses, the use of porcine tissue enabled us to gather data from a more homogenous group of animals, collecting and culturing tissue from multiple animals at one time. However, despite certain structural similarities between the gastric mucosa of pigs and horses, there exist considerable morphological differences in the mammalian GI tract across species. Diet is known to play a considerable role in influencing GI morphology, and this represents a meaningful difference between pigs, an omnivorous species, and horses, which are strict herbivores. Therefore, the use of porcine tissue in this study does represent a limitation for the direct translation of the results to horses, and as such, the results should be interpreted with caution. However, this may mainly be reflected in the direct scale of the response as opposed to the nature of tissue response to the culture conditions and gastric digests. Nevertheless, direct comparative studies between the secretory response of porcine and equine tissue to physiological stimuli such as Ach would strengthen conclusions, which could be drawn across species.

## Conclusion

The inclusion of acidic gastric digests and the cholinergic agonist carbachol did not alter cell viability as measured by calcein AM/Eth-D staining in porcine antral mucosa cultured over 72 h. The creation of an acidic environment through the addition of a blank gastric digest to the media eliminated cholinergic gastrin stimulation and enhanced NO secretion into the media. The addition of G's Formula to the acidic digest maintained the tissue response to cholinergic stimulation such that gastrin secretion was increased over time and media nitrite demonstrated the same profile as tissue cultured in a neutral environment. However, future studies would benefit from histological analysis and by including measures of mucosal activity and defense such as acid or mucus production and protein metabolism. The functional significance of the *in vitro* effect of G's Formula also needs to be verified and would benefit from *in vivo* research.

## Data Availability Statement

The raw data supporting the conclusions of this article will be made available by the authors, without undue reservation.

## Ethics Statement

Ethical review and approval was not required for the animal study because postmortem abattoir tissue was utilized.

## Author Contributions

JM: experimental design, data collection, data analysis, manuscript composition, and manuscript editing. WP: experimental design and manuscript editing. Both authors contributed to the article and approved the submitted version.

## Funding

This research was funded by G's Organic Solutions INC. (Duncan, BC, CAN) (Grant number 053305). JM is funded by NSERC. The funder was not involved in the study design, collection, analysis, interpretation of data, the writing of this article, or the decision to submit it for publication.

## Conflict of Interest

The authors declare that the research was conducted in the absence of any commercial or financial relationships that could be construed as a potential conflict of interest.

## Publisher's Note

All claims expressed in this article are solely those of the authors and do not necessarily represent those of their affiliated organizations, or those of the publisher, the editors and the reviewers. Any product that may be evaluated in this article, or claim that may be made by its manufacturer, is not guaranteed or endorsed by the publisher.
