# Supplementary material for: Gastrin and Nitric Oxide Production in Cultured Gastric Antral Mucosa Are Altered in Response to a Gastric Digest of a Dietary Supplement
Source: Front Vet Sci. 2021 Oct 4;8:684203. doi: 10.3389/fvets.2021.684203 (PMC8520902; doi:10.3389/fvets.2021.684203)
Supplement: Supplementary file 1 [file Data_Sheet_1.DOCX]

Supplementary Data

Gastrin concentrations not standardized per g of tissue wet weight

| Treatment | Hour | Gastrin(pg/ml) | SEM |
| --- | --- | --- | --- |
| CO_stim | 48 | 376 | 37.6 |
|  | 60 | 377 | 37.6 |
|  | 72 | 462 | 37.6 |
|  |  |  |  |
| CO_non | 48 | 387 | 39.4 |
|  | 60 | 316 | 39.4 |
|  | 72 | 374 | 41.5 |
|  |  |  |  |
| BL_stim | 48 | 411 | 36.1 |
|  | 60 | 327 | 36.1 |
|  | 72 | 399 | 36.1 |
|  |  |  |  |
| BL_non | 48 | 373 | 34.6 |
|  | 60 | 344 | 34.6 |
|  | 72 | 364 | 34.6 |
|  |  |  |  |
| DF_stim | 48 | 365 | 36.1 |
|  | 60 | 428 | 36.1 |
|  | 72 | 451 | 36.1 |
|  |  |  |  |
| DF_non | 48 | 405 | 41.6 |
|  | 60 | 438 | 41.6 |
|  | 72 | 422 | 41.6 |

Table 2. Media gastrin (pg/ml) as assessed by spectrophotometric assay of a commercial ELISA for porcine gastrin over time in porcine antral explants stimulated with either 10^-5^ M carbachol (_stim) or sterile PBS (_non). Explants were taken with 4mm punch biopsy and cultured in sterile DMEM containing 100 U/L penicillin and 100 µl/L streptomycin, 1 ml/L amphotericin, 5% fetal bovine serum that was refreshed daily. CO_stim (n=10)/CO_non (n=10), explants treated with sterile PBS. BL_stim (n=10)/BL_non (n=13), explants treated with a blank gastric digest. DF_stim (n=11)/DF_non (n=9), explants treated with a simulated gastric digest of G’s Formula^TM^, composed of dried cabbage, carrot, hemp, and oat flour.
